# Spatial and Temporal Examination of Bivalve Communities in Several Estuaries of Southern California and Northern Baja California, MX

**DOI:** 10.1371/journal.pone.0148220

**Published:** 2016-02-03

**Authors:** Anai Novoa, Theresa S. Talley, Drew M. Talley, Jeffrey A. Crooks, Nathalie B. Reyns

**Affiliations:** 1 Department of Environmental and Ocean Sciences, University of San Diego, San Diego, California, United States of America; 2 Department of California Sea Grant Extension Program, Scripps Institution of Oceanography, University of California San Diego, La Jolla, California, United States of America; 3 Department of Tijuana River National Estuarine Research Reserve, Imperial Beach, California, United States of America; The Evergreen State College, UNITED STATES

## Abstract

A combination of historical bivalve surveys spanning 30–50 years and contemporary sampling were used to document the changes in bivalve community structure over time at four southern California and one northern Baja California estuaries. While there are limitations to the interpretation of historic data, we observed generally similar trends of reduced total bivalve species richness, losses of relatively large and/or deeper-dwelling natives, and gains of relatively small, surface dwelling introduced species across the southern California estuaries, despite fairly distinct bivalve communities. A nearly 50-year absence of bivalves from two wetlands surveyed in a Baja California estuary continued. A combination of site history and current characteristics (e.g., location, depth) likely contributes to maintenance of distinct communities, and both episodic and gradual environmental changes likely contribute to within-estuary temporal shifts (or absences). We highlight future research needed to determine mechanisms underlying patterns so that we can better predict responses of bivalve communities to future scenarios, including climate change and restoration.

## Introduction

Estuarine bivalve communities have been altered through direct and indirect human effects, including overharvesting, habitat loss and alteration, pollution, the invasion of introduced species and climate change [1,2,3,4,5]. Within southern California USA and Baja California, Mexico, coastal ecosystems are particularly impacted by heavy use, coastal development, and associated stresses, all of which have potentially influenced bivalve community structure. Indeed, sporadic studies over the past 50 years of bivalve communities within estuaries throughout this region indicate dramatic local shifts in intertidal bivalve communities [2,6] (and references therein). These community-level changes have likely resulted in concomitant changes in the ecosystem functions and services, such as water filtration, biodeposition, bioturbation [7,8], provision of substrate for epibionts (e.g., algae and barnacles), refugia (e.g., crabs, fishes)[9], and food resources for economically-important and threatened species (e.g., fish, [10]; crabs, [11]; birds, [12]). Data that can inform predictions about changes in bivalve communities, and their services, is of particular interest as we move into an era of changing climates and environmental disturbances.

A combination of contemporary and historical data helps us to understand the current state of ecosystems in the context of long time frames, and can shed light on the potential anthropogenic and environmental influences on community dynamics [13]. Although historical data have a variety of potential limitations, including, inconsistent collection methods and potential biases in temporal and spatial coverage, they are all we have to characterize past communities [13,14] and serve as a valuable benchmark against which current and future changes can be assessed [15,16,17]. In this study, we therefore combined historical datasets with contemporary sampling in four southern California and one northern Baja California estuaries to document the changes in (1) the diversity and abundance, and (2) structure of intertidal bivalve communities within and between estuaries over a nearly 50 year time period. Patterns were compared to a null hypothesis of similar, random shifts in bivalve communities across all estuaries through time. In the late-1960s to early-1970s, studies of intertidal bivalve communities were conducted throughout estuaries in southern California (Mugu Lagoon, Los Peñasquitos Lagoon, Mission Bay, Tijuana River Estuary) [18,19,20,21] and northern Baja California (e.g., Bahía de San Quintín) [20]. There were a few bivalve studies conducted in some of these estuaries in the intervening years helping to fill in the time line, and our current regional assessments document the present status of bivalve communities.

## Materials and Methods

Study estuaries were chosen based on availability of historic data and distribution throughout the region (Table 1). Patterns of bivalve community change *within* each estuary were documented by comparing recently collected bivalve data (this study) to those from previous studies of the same estuary (Table 1). Regional bivalve community trends were documented by comparing bivalve community data (both previous and from this study) across all estuaries. Some previous studies included detailed maps, which allowed us to precisely re-sample the locations, and others provided a description where we could broadly relocate original sampling locations.

**Table 1:** Study site, sample year, latitude/longitude (if available), core and sieve size used, location reported, and reference for past surveys. **(**“---”refers to unavailable data).

Site	Sample Year	Latitude/ Longitude	Core size (cm) Diam. x Depth	Mesh size (mm)	No. samples (cores) per sampling effort	No. sampling efforts	Location reported by study	Reference
Mugu Lagoon	1966	34°06’14”/ 119°05’58”	25 x 25	1	15	1	Description	[20]
Mugu Lagoon	1969–1972	34°06’/ 119°05’	28 x 56	3.2	19–74	10	Description	[21]
Mugu Lagoon	2013	34°06’16”/ 119°05’33”	10 x 20	1	60 (5 subsamples x 12 sites)	1		Current study [22]
Los Peñasquitos Lagoon	1964	32°56’2”/ 117°15’24”	---	---		---	Map	[18]
Los Peñasquitos Lagoon	1967–1968	32°56’41”/ 117°15’11”	30 x 30	---	13	7	Map	[19]
Los Peñasquitos Lagoon	1987–2008	32°56’37”/ 117°15’21”	15 x 20	3	9–18	38	Map	[6], PERL, TRNERR (unpub.)
Los Peñasquitos Lagoon	2013	32°55’59”/ 117°15’32”	10 x 20	1	36 (9 subsamples x 4 sites)	1		Current study [22]
Mission Bay	1964–1966	32°47’33”/ 117°14’06”	25 x 25	1	12 (2 subsamples x 6 sites)	5	Description	[20]
Mission Bay	1994–1996	32°47’29”/ 117°14’24”	25 x 25	1	12 (2 subsamples x 6 sites)	4	Map	[2]
Mission Bay	2009–2011	32°47’27” 117°13’39”	0.0625 m^2^	1	12 (2 subsamples x 6 sites)	3	---	Reyns (unpub.)
Mission Bay	2012–2013	32°47’27”/ 117°13’39”	15 x 30	1	12 (2 subsamples x 6 sites)	2		Current study [22]
Tijuana River Estuary	1969–1972	32°33’/ 117°08’	28 x 56	3.2	19–74	10	Description	[21]
Tijuana River Estuary	1976	32°33’18”/ 117°07’14”	13.5 x 40	2	117		Map	[23]
Tijuana River Estuary	1986–1987	32°33’27”/ 117°07’23”	15 x 25	---	30–45		Map	[24]
Tijuana River Estuary	1986–2007	32°34’29”/ 117°07’24”	15 x 20	3	18–24	39	Map	[6], PERL, TRNERR (unpub.)
Tijuana River Estuary	2013	32°34’04”/ 117°07’53”	10 x 20	1	60 (5 subsamples x 12 sites)	1		Current study [22]
Bahía de San Quintín	1966	30°30’52”/ 116°00’39”	25 x 25	1	4–10	1	Description	[20]
Bahía de San Quintín	2004	---	10 x 50	5	---	---	---	[25,26]
Bahía de San Quintín	2013	30°29’52”/ 116°00’04”	15 x 30	1	24 (2 subsamples x 12 sites)	1		Current study [22]

### Sampling stations and protocol

**Fig 1:**
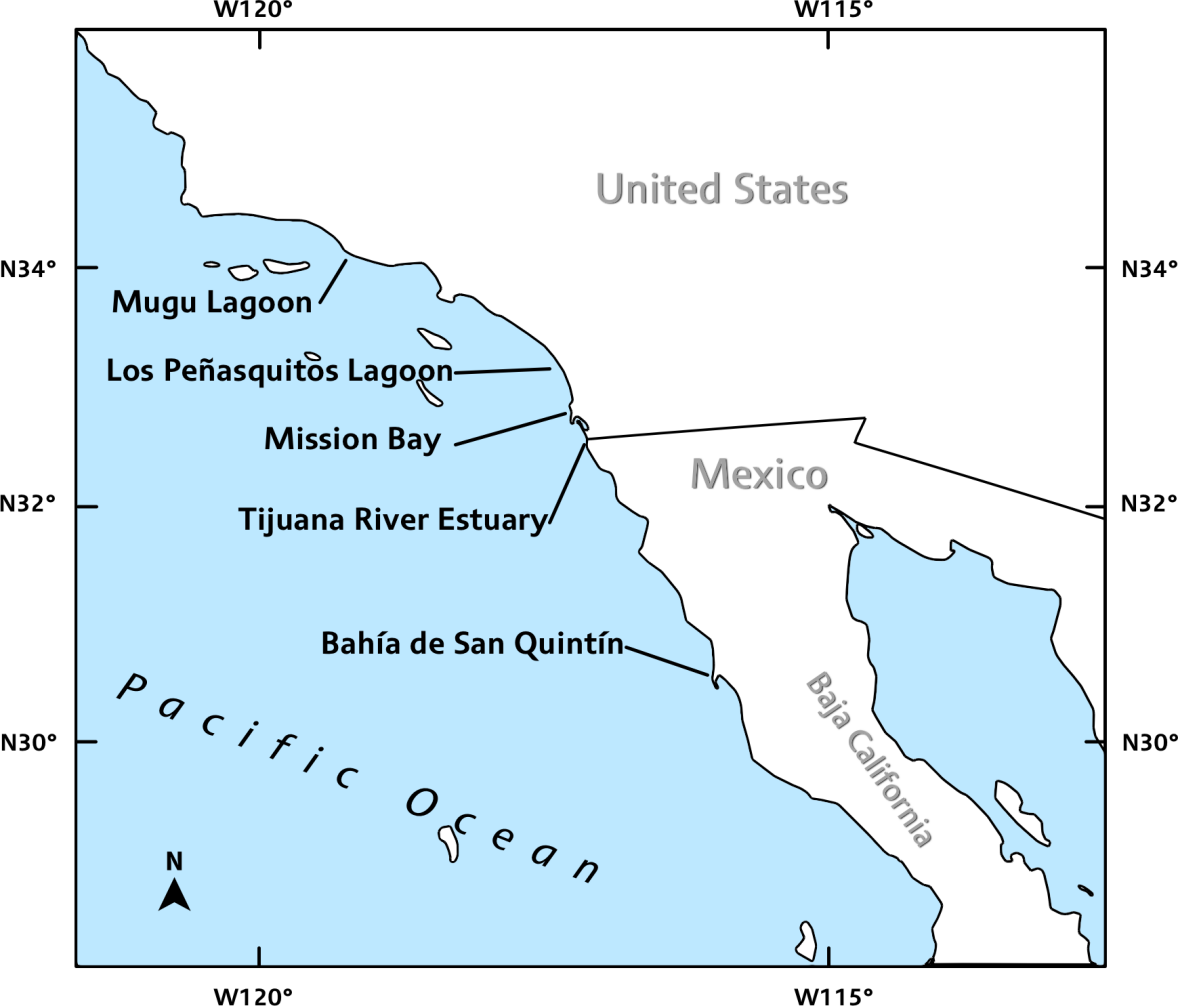
Map of intertidal study sites in southern California, USA and northern Baja California, MX. The map was adapted from a Creative Commons 3.0 image from Wikimedia Commons.

Intertidal salt marsh creeks were sampled within five estuaries located throughout southern California and northern Baja California, including Mugu Lagoon, Los Peñasquitos Lagoon, Mission Bay, and Tijuana River Estuary in California, USA and two distinct wetlands within Bahía de San Quintín, Baja California, Mexico (Tables 1 and 2; Fig 1). Contemporary sampling spanned seasons and weather patterns, however there were no catastrophic weather events (e.g., storms, estuary closures) during this time span (December 2012- September 2013; see below). All of the bivalve species sampled were either post-settlement juveniles or adults (1 mm or greater in size) that are characteristically long-lived, thus weather patterns during the sampling period should not have greatly influenced the compositions observed.

**Table 2:** Morphological characteristics of study sites. Pore water salinity was collected on April 2013 in Mission Bay, June 2013 in Bahía de San Quintín, January 2014 in Tijuana River Estuary, February 2014 at Mugu Lagoon and Los Peñasquitos Lagoon.

Site	Total area of estuary (km^2^)	Total area of tidal flat (km^2^)	Area of salt marsh/channel habitat (km^2^)	% Pore water salinity (± SD)	Reference:
**Mugu Lagoon**	10.12	0.52	0.15	27 (±0.6)	[27]
**Los Peñasquitos Lagoon**	1.42	.0.13	0.12	26.25 (±2.39)	[28,29]
**Mission Bay**	18.62	0.13	0.46	37 (±0.93)	[30]
**Tijuana River Estuary**	10.12	0.60	2.49	27.33 (±0.7)	[28]
**Bahía de San Quintín**	48.00	8.85	9.57	31.58 (±3.28)	[31]

Permission to enter the Tijuana River National Estuarine Research Reserve was granted by Brian Collins, US Fish and Wildlife Service under Special Use Permit 81680 14002. The Mission Bay site fell within the boundaries of the University of California Kendall Frost Marsh Reserve and permission to conduct the sampling was granted from Isabelle Kay, Reserves Manager at the U.C. Natural Reserve System. Entrance into Mugu Lagoon occurred as part of the San Onofre Nuclear Generating Station (SONGS) Mitigation Monitoring team led by S. Schroeter and H.M. Page. Collections were conducted under the California Fish and Wildlife Scientific Collection Permit administered to T.S. Talley (Permit number SC-5295).

Fieldwork was conducted as close as possible to the time of year that previous samples were taken, so that results would be temporally comparable (Tables 1 and 2; Fig 1). Samples were collected in September 2013 from Mugu Lagoon, Los Peñasquitos Lagoon, and Tijuana River Estuary; in December 2012 and April 2013 at Mission Bay (Kendall-Frost Marsh Reserve/Northern Wildlife Preserve), and in June 2013 at North Marsh and Cemetery Marsh, both in Bahía de San Quintín. A station represents the location of the bivalve core samples within the site, which were taken between 30-cm above and below Mean Lower Low Water. The number of stations varied between sites (Table 1).

The intertidal marsh site surveyed within Mugu Lagoon is located approximately 0.4 km from the mouth of the estuary (Table 2; Fig 1). We sampled 12 stations, which consisted of 6 stations located in the main channel and another 6 stations located in the side creeks. At each station, five bivalve subsamples were taken, each separated by approximately 10 meters yielding a total of 60 samples. Sampling areas within Mugu Lagoon overlapped with most of the areas sampled in previous studies.

In the Los Peñasquitos Lagoon site, four stations were sampled each containing 9 bivalve subsamples taken 3–5 m apart (total of 36 samples) (Table 2; Fig 1). The sampling areas were located from the mouth to the tidal creeks in the eastern end of the lagoon and spatially overlapped with all of the previous studies except for [6]. Desmond et al. (2002) sampled all stations except for a station located in the southern end of Los Peñasquitos Lagoon, which was taken into account when analyzing data.

The tidal creek surveyed within the Mission Bay site (Kendall-Frost Marsh Reserve/North Wildlife Preserve, hereafter referred to as Mission Bay) is about 5 km from the mouth and is located at the northeast corner of the bay (Table 2; Fig 1). We sampled six stations, each containing two bivalve subsamples taken 2–3 m apart (total of 12 bivalve samples). Stations were located equidistantly along the wetland’s main channel. This site had similar sampling procedures and overlaps with all of the previous studies.

The intertidal marsh site at Tijuana River Estuary is located approximately 0.6 km from the mouth of the estuary (Table 2; Fig 1). We sampled 12 stations, which consisted of 6 stations located in the main channel and another 6 stations located in the side creeks. At each station, five subsamples were taken, each separated by approximately 10 meters yielding a total of 60 samples. Sampling areas within this site overlapped with most of the areas sampled in previous studies. The south arm of Tijuana River Estuary sampled by Peterson [21] has since changed significantly due to sedimentation, so no samples were collected there and thus this dataset were interpreted with caution.

Two distinct sites were sampled for bivalves in Bahía de San Quintín: Cemetery Marsh (30°27’34” N, 115°56’05” W) located about 7.4 km from the mouth, and North Marsh (30°29’53” N, 116°00’04” W) located 14.8 km from the mouth of the estuary (the sampling locations in MacDonald 1969) (Table 2; Fig 1). At each site, six stations were sampled, each containing a pair of subsamples taken 2–3 m apart (total of 12 samples at each site).

Bivalve core size varied by site as a result of leveraging different ongoing sampling programs in these areas. Mission Bay and Bahía de San Quintín stations had 15-cm diameter x 30-cm depth cores. Mugu Lagoon and the Tijuana River Estuary are part of current monitoring of San Onofre Nuclear Generating Station (SONGS) mitigation program, and a bivalve core size of 10-cm diameter x 20-cm depth was used in this study. A 10-cm diameter x 20-cm core was also used at Los Peñasquitos Lagoon. Bivalve cores were each wet sieved in the field using a 1-mm mesh sieve, but sieve sizes vary between this and prior studies (Table 1) thus data were examined with caution since the ranges of sieve size used may influence comparison of total abundance (e.g., not capturing juveniles). The portion of sample retained on the sieve was collected and frozen at -18°C until bivalve enumeration and identification could be performed under a dissecting microscope in the lab.

### Data analyses

Comparisons of bivalve abundances across sites (within time periods) and across time periods (within sites) were made using one-way ANOVA and Tukey’s HSD a-posteriori pairwise comparisons in JMP Pro 12.0.1 [32]. The average total bivalve abundance per study, standardized to a 0.25m^2^ diameter core, was used as the dependent variable. Abundance data were log10 (x + 1) transformed prior to statistical analyses to normalize the data structure and homogenize the variances [33].

Comparisons of species richness within and among estuaries over time were made using sample-based rarefaction curves created in EstimateS [34]. Using our sample-based abundance data, the individuals within samples were randomly resampled, without replacement, to calculate the expected number of species and unconditional confidence intervals [35] for a given number of individuals (within samples). Estimates are based on the number of species averaged across resampling runs of each sample. Random starting points for 100 runs were used, with the upper abundance limit of 10 set for rare species [34]. Our species richness data were the abundance of each species averaged across all the samples collected during a sampling event (sampling of a site within a distinct study period). Differences in species richness within estuaries over time was determined by visual inspection of the 95% confidence intervals, where non-overlapping intervals indicate differences.

Changes in bivalve communities among sites over time were explored using multivariate statistical analyses in which the averaged bivalve abundance data were grouped into (used as replicates in) four broad time periods: late 1960s-mid 1970s, late 1980s-mid 1990s, late 1990s-mid 2000s, and late 2000s-present. The time period groupings were defined based on little to no bivalve community differences between years within these periods (ANOSIM p ≥ 0.16) or, when only one survey per year was available, by visual comparisons that revealed no consistent changes in species abundances.

Bivalve community similarities and differences were visualized using non-metric multidimensional scaling (nMDS; see [36]) on Bray-Curtis similarity indices of log (x+1) transformed, unstandardized data using R Statistical Platform [37]. Six different random starting points with up to 1,000 steps were used. The stress values from the six runs were examined for stability to determine whether a global solution had been found. Only analyses with stress values of <0.2 were used; stress is a measure of how well the solution (in this case the two-dimensional nMDS plots) represents the distances between the data. Clarke (1993) suggests values <0.1 are good and <0.2 are useful. Significance testing for differences in bivalve composition among estuaries and among decadal time periods was completed using an analysis of similarity (ANOSIM) procedure [36]. The significance levels of resultant pairwise comparisons were determined using sequential Bonferroni-adjusted alphas. Testing for interactions between time period and estuary was not possible due to the lack of replication (studies) during some time periods for some estuaries so analyses were run separately. Analyses of bivalve dissimilarities between site and date groups, and the particular taxa contributing to the dissimilarity, were carried out using SIMPER [36]. The SIMPER results specify which taxa are responsible for the ANOSIM results by comparing the average abundances of taxa between groups. The average dissimilarity between samples from the groups is computed and then broken down into contributions from each species. Those species with high average terms relative to the standard deviation are important in the differentiation of groups.

There were no live bivalves present in Bahía de San Quintín during any of the sampling years (1966, 2004, and 2013) so these sites were removed from both the within- and among-site comparisons.

## Results

### Trends in abundance

**Fig 2:**
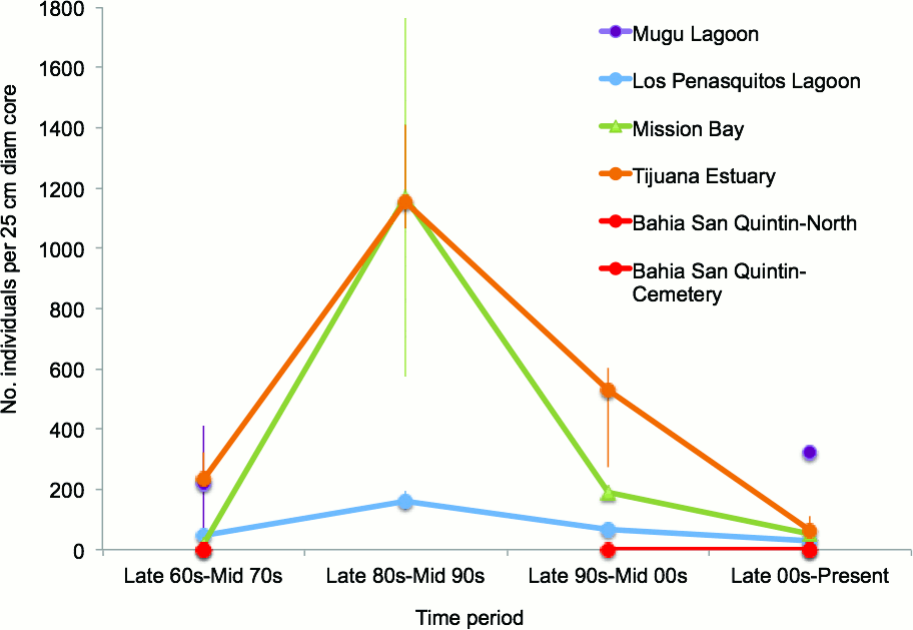
Historical comparison of average bivalve abundances for each estuary studied in southern California (Mugu Lagoon, Los Peñasquitos Lagoon, Mission Bay, Tijuana River Estuary) and northern Baja California (Bahía de San Quintín). **Error bars are ±1 S.E.** Bivalve species abundance data were averaged by year and grouped into four broad time periods.

Three of the six estuaries had unimodal abundance patterns through time, with abundance peaks over 6x greater in Mission Bay and Tijuana Estuary than Los Peñasquitos Lagoon, in the late 80s-mid 90s (Table 3A and 3B; Fig 2). Abundances tended to be lowest in the most recent (late 2000s –present) and/or the earliest time period (late 1960s-mid 1970s) (Table 3A; Fig 2). Abundances in the two Bahía de San Quintín sites were consistently zero and no data existed for Mugu from the late 1980s to the mid 2000s.

**Table 3:** Results of comparisons of total bivalve abundance across (A) time periods and (B) estuaries using one-way ANOVA and Tukey’s HSD pairwise tests. Abundances used were the average total abundance reported from each study that was used in this study. ML = Mugu Lagoon, LPL = Los Peñasquitos, MB = Mission Bay, TJE = Tijuana Estuary, --- = no significant difference detected.

**A. Comparison of abundances across estuaries (within time period)**				
**Time Period**	**P**	**df (n)**	**F**	**Pairwise results**
Late 60s-mid 70s	0.614	3,3 (7)	0.7	---
Late 80s-mid 90s	0.006	2,18 (21)	6.8	MB, TJE > LPL
Late 90s-mid 00s	<0.0001	2,17 (20)	21.4	TJE ≥ MB ≥ LPL
Late 00s-mid 10s	0.004	3,8 (12)	10.2	ML > LPL, MB, TJE
**B. Comparison of abundances across time periods (within sites)**				
**Site**	**P**	**df (n)**	**F**	**Pairwise results**
Mugu Lagoon	0.800	1,1 (3)	0.1	---
Los Peñasquitos Lagoon	0.017	3,21 (25)	4.25	80s-90s ≥ 60s-70s ≥ 90s-00s, 00s-10s
Mission Bay	0.045	3,5 (9)	5.72	80s-90s ≥ 90s-00s ≥ 60s-70s, 00s-10s
Tijuana River Estuary	0.34	3,19 (23)	3.7	80s- 90s > all others

### Trends in diversity

**Fig 3:**
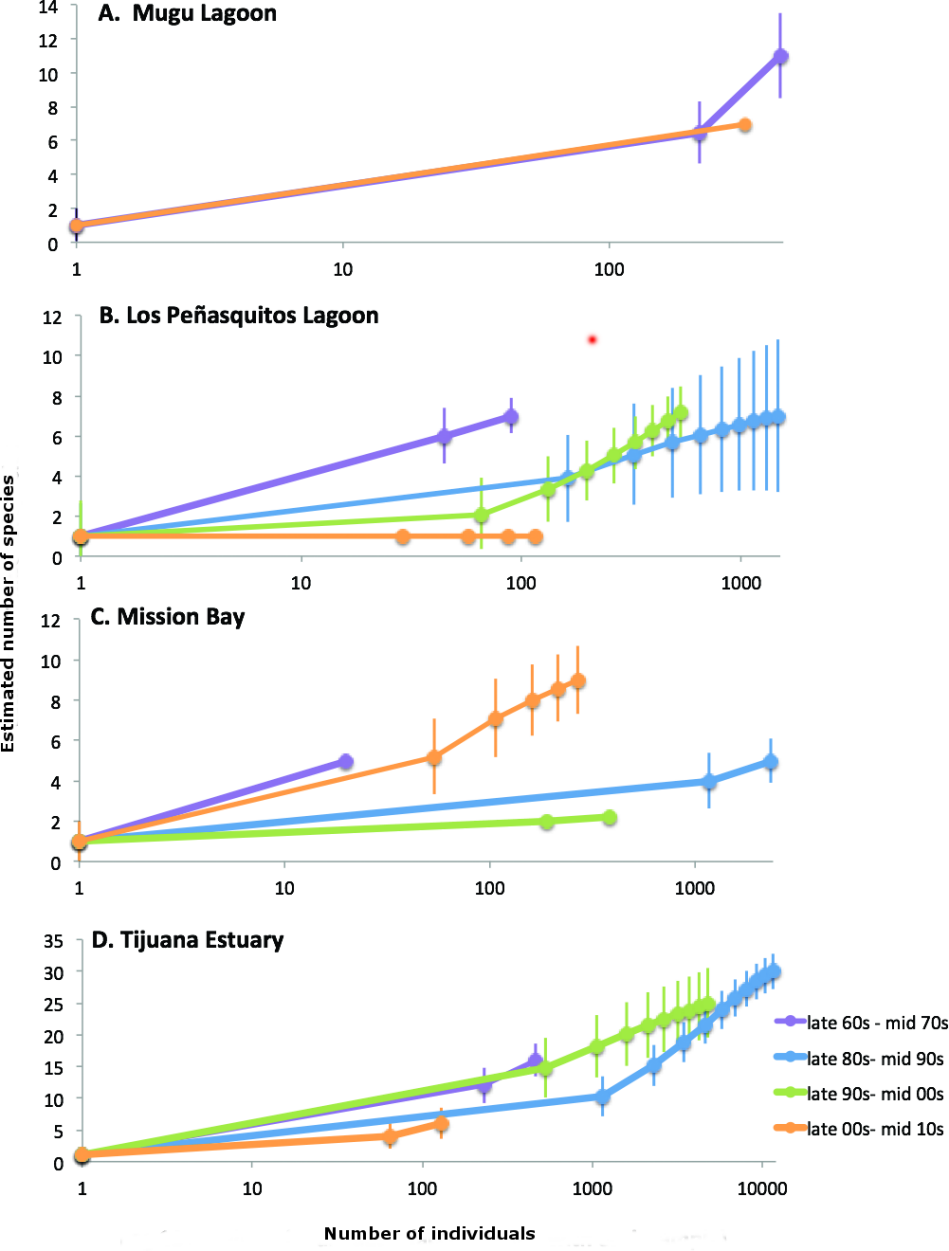
Sample-based rarefaction curves (species richness as a function of number of individuals per sample) (A-D) for each southern California estuary studied: Mugu Lagoon, Los Peñasquitos Lagoon, Mission Bay, and Tijuana River Estuary. **Error bars represent 95% confidence intervals.** Rarefaction data were averaged by year and grouped into 4 broad time periods.

Bivalve richness, as measured by sample-based rarefaction, was similar or higher in the late 60s-mid 70s than in all other time periods (Fig 3A–3D). Richness throughout the late 80s to mid 00s tended to be lower than in the 60s, and either higher or lower than current levels depending upon estuary (Fig 3B–3D). Current richness levels were generally similar to the late 60s-mid 70s except for Los Peñasquitos Lagoon, where only one species was found as compared to 7–8 in earlier time periods (Fig 3B). Similarly, the total number of species per estuary indicated declines in most other estuaries, too. Mugu Lagoon had 11 species in the late 1960s-mid 1970s, but 7 species in the most recent time period. Tijuana River Estuary had a total of 16 species in the late 1960s-mid 1970s, 25–29 throughout the late 1980s to the mid 2000s, and then six in the most recent time period. Mission Bay was the exception with the highest species richness in the most recent time period (9 species total), the lowest in the late 1990s-mid 2000s (2 species), and five species during the late 1960s-mid 1970s and the late 1980s-mid 1990s time periods. The two wetlands in Bahía de San Quintín had no live bivalves throughout the study period.

### Trends in bivalve community composition

#### Between-estuaries

Communities differed between each of the decadal time periods (ANOSIM Global R = 0.43, P = 0.001) (Table 4A). The biggest difference occurred between communities found in the late 60s-mid 70s and all other time periods (69–70% dissimilarity) (Table 4A). Communities in the late 80s-mid 90s, when abundances of the introduced *Musculista (= Arcuatula) senhousia* peaked along with increases in *Mytilus galloprovincialis*, *Tagelus californianus*, *Macoma nasuta* and *Leukoma staminea*, and those in the late 90s-mid 00s, when these species declined but were still abundant, were most similar (47% dissimilarity) (Table 4A). The late 80s to mid 00s also corresponded with the decline or loss of many natives including *Laevicardium substriatum*, *Macoma* sp., *Leukoma laciniata*, *Nuttallia nuttallii*, *Tresus nuttallii*, and *Cryptomya californica* (Table 4A; S1 Table). The most recent communities, compared with those in the late 60s-mid 70s, continued to support higher abundances of *M*. *senhousia* and lower abundances of the natives that declined throughout the 1980s and 1990s (Table 4A; S1 Table). The most recent communities (late 00s-mid 10s) were 57–62% different from those occurring between the late 80s to the mid 00s (Table 4A) owing to further declines of *M*. *senhousia*, *T*. *californianus*, *L*. *staminea*, *M*. *nasuta*, and higher abundances of a more recent introduced species, *Venerupis philippinarum* (Table 4A; S1 Table).

**Table 4:** Comparisons of bivalve assemblages (abundance of each species) between (A) decadal time periods and (B) four southern California estuaries. Bivalve data were averaged by year for each estuary (within each study) and pooled into four decadal time periods. Shown are ANOSIM pairwise p values, and both the % similarity and the species contributing ≥ 5% of the variability between groups from the SIMPER analyses. ANOSIM Global test statistics are: (A) Global R = 0.43, P = 0.001, and (B) Global R = 0.63, P = 0.001.

**A. Time periods**	**ANOSIM Pairwise p**	**Dissimilarity (%)**	**Species contribution (%) for those contributing ≥4%**
Late 60s-mid 70s vs. Late 80s-mid 90s	0.001	70	*M*. *senhousia* (11), *T*. *californianus* (10), *L*. *substriatum* (9), *L*. *staminea* (9), *Macoma* sp. (8), *M*. *galloprovincialis* (6), *M*. *nasuta* (6), *L*. *laciniata* (5), *N*. *nuttallii* (5)
Late 60s-mid 70s vs. Late 90s-mid 00s	0.001	69	*Macoma* sp. (15), *L*. *substriatum* (12), *T*. *californianus* (9), *M*. *galloprovincialis* (9), *L*. *lacinata* (8), *M*. *senhousia* (6), *L*. *staminea* (6), *T*. *nuttallii* (5)
Late 60s-mid 70s vs. Late 00s-mid 10s	0.015	69	*Macoma* sp. (13), *L*. *substriatum* (11), *T*. *californianus* (8), *C*. *californica* (7), *M*. *nasuta* (6), *M*. *galloprovincialis* (6), *L*. *laciniata* (6), *L*. *staminea* (5), *V*. *philippinarum* (5), *N*. *nuttallii* (5)
Late 80s-mid 90s vs. Late 90s-mid 00s	0.001	47	*T*. *californianus* (17), *M*. *senhousia* (13), *L*. *staminea* (12), *M*. *nasuta* (9), *Laevicardium* sp. (8), *Tellina* sp. (6)
Late 80s-mid 90s vs. Late 00s-mid 10s	0.001	62	*M*. senhousia (22), *T*. *californianus* (16), *L*. *staminea* (15), *L*. *substriatum* (5)
Late 90s-mid 00s vs. Late 00s-mid 10s	0.051	57	*T*. *californianus* (25), *M*. *senhousia* (15), *L*. *staminea* (12), *Laevicardium* sp. (5), *Chione* sp. (5), *Tellina* sp. (5)
**B. Estuaries**			
Mugu Lagoon vs. Los Peñasquitos Lagoon	0.083	87	*L*. *staminea* (15), *Macoma* sp. (14), *M*. *nasuta* (12), *C*. *californica* (12), *T*. *californianus* (8), *L*. *substriatum* (7), *M*. *secta* (7), *L*. *laciniata* (5)
Mugu Lagoon vs. Mission Bay	0.167	71	*T*. *californianus* (13), *L*. *staminea* (13), *C*. *californica* (12), *M*. *senhousia* (11), *M*. *nasuta* (11), *Macoma* sp. (10), *M*. *secta* (8), *C*. *fluctifraga* (8)
Mugu Lagoon vs. Tijuana River Estuary	0.889	52	*C*. *californica* (14), *M*. *nasuta* (12), *T*. *californianus* (11), N. nuttallii (9), *M*. *secta* (7), *L*. *substriatum* (6), *T*. *carpenteri* (5)
Los Peñasquitos Lagoon vs. Mission Bay	0.014	66	*M*. *senhousia* (37), *T*. *californianus* (18), *M*. *nasuta* (9), *V*. *philippinarum* (8), *L*. *staminea* (6)
Los Peñasquitos Lagoon vs. Tijuana River Estuary	0.001	69	*L*. *staminea* (17), *M*. *nasuta* (10), *M*. *senhousia* (8), *L*. *substriatum* (7), *T*. *carpenteri* (6), *T*. *californianus* (6), *C*. *californica* (5)
Mission Bay vs. Tijuana River Estuary	0.001	69	*L*. *staminea* (19), *M*. *senhousia* (12), *T*. *californianus* (9), *M*. *nasuta* (7), *L*. *substriatum* (5), *V*. *philippinarum* (5)

**Fig 4:**
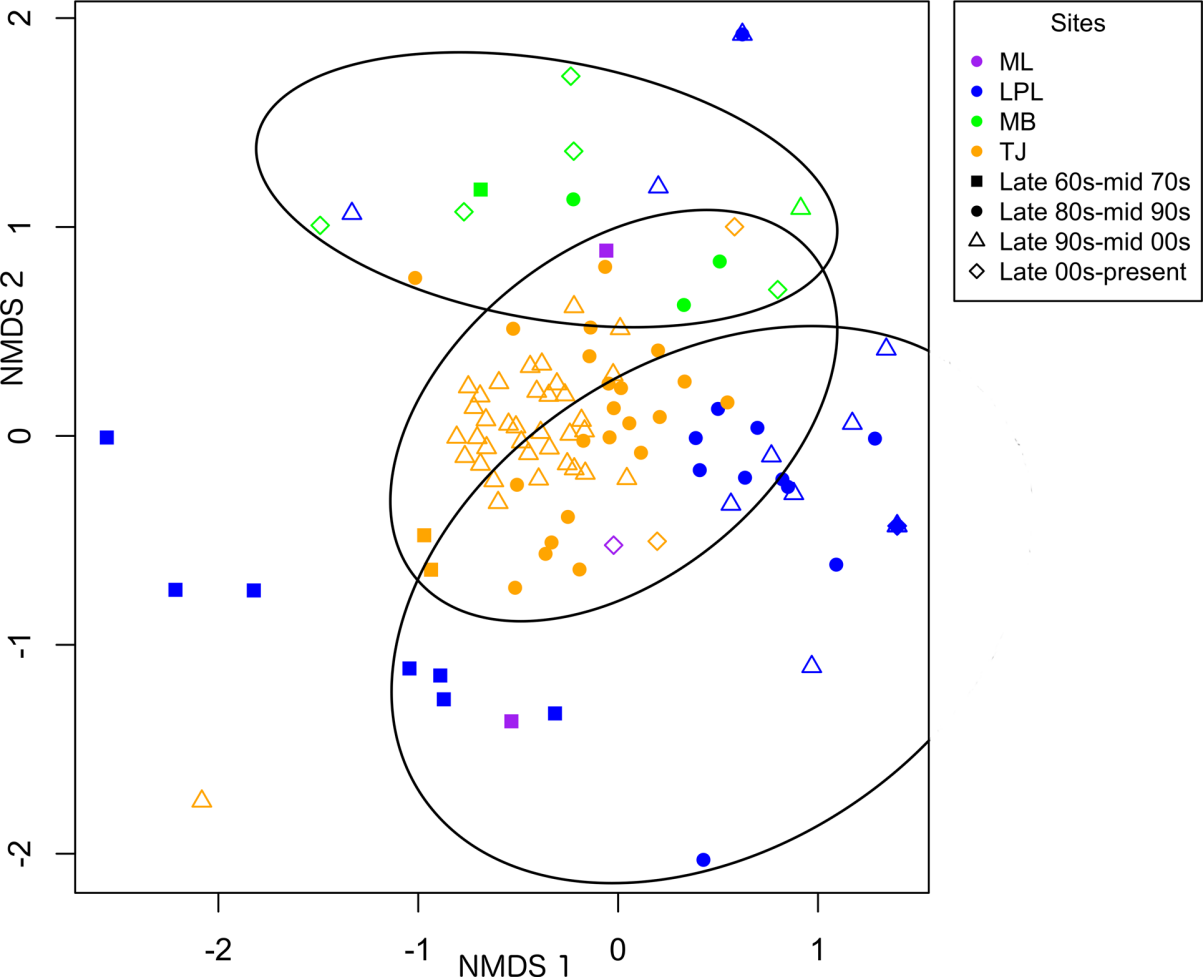
Non-metric multidimensional scaling plot of bivalve abundances over time for each southern California estuary studied: Mugu Lagoon (ML), Los Peñasquitos Lagoon (LPL), Mission Bay (MB), and Tijuana River Estuary (TJ). Stress value = 0.17. Bivalve surveys were averaged by year and grouped by decadal time period. Ellipses are drawn using visual assessment of the clustering of points within a Site-Time Period group.

Bivalve communities were generally distinct between estuaries (ANOSIM Global R = 0.63, P = 0.001) despite community changes within the estuaries with time (Fig 4). Mugu Lagoon bivalve communities were distinct from Los Peñasquitos and Mission Bay (71–87% dissimilarity) due to higher abundances of a diversity of natives including *L*. *staminea*, *M*. *nasuta*, *Macoma* sp., *Macoma secta*, *C*. *californica*, and *N*. *nuttallii* (Fig 4; Table 4B; S1 Table). These pairwise comparisons were, however, not significant at p = 0.05 but this was likely in part due to the small sample size available for Mugu Lagoon (3 studies total). Tijuana River Estuary most resembled Mugu Lagoon (p = 0.89, 52% dissimilarity) (Table 4B) and was distinct from Los Peñasquitos and Mission Bay (69% dissimilarity each) (Table 4B; Fig 4) due to generally higher abundances of the same natives that made Mugu distinct, as well as *Tellina carpenteri*, *L*. *substriatum*, *T*. *californianus* and the invasive *M*. *senhousia* (Table 4B). Los Peñasquitos Lagoon communities were generally distinct from other estuaries due to dominance of *T*. *californianus* in most time periods (Fig 4; Table 4B; S1 Table). Mission Bay bivalve communities were distinct due to consistently higher densities of the introduced species *M*. *senhousia* and *V*. *philippinarum*, and the native *Chione fluctifraga* (Fig 4; Table 4B; S1 Table).

#### Within-estuary

Mugu Lagoon. Of the 11 species present in the late 1960s-mid 1970s, only five species were found in the late 2000s-present. Missing were mostly the larger species, such as *Diplodonta orbellus*, *N*. *nuttallii*, *Saxidomus nuttalli* and *T*. *nuttallii*. Of the five species still found, *Leukoma staminea* and *Cryptomya californica* experienced a 30–70% decrease in abundance (S1 Table); *C*. *californica* had been the most abundant bivalve in the early time period (S1 Table). *Macoma secta*, *M*. *nasuta*, and *T*. *californianus*, however, were 2–19% more abundant in the most recent time than the early period, with *T*. *californianus* the most abundance species recently (175 individuals per 490 cm^2^) (S1 Table).

For the two time decadal periods available for Mugu Lagoon, 12 native bivalve species and one introduced species were found (S1 Table). The Manila clam, *V*. *philippinarum* was documented for the first time, albeit in relatively low abundance (1.4 individuals per 490cm^2^), near the center of the main channel in Mugu Lagoon during 2013 sampling. Additional Manila clams were also observed during this 2013 sampling period, between sampling locations.

Los Peñasquitos Lagoon. During the early 1960s, live bivalves were absent, corresponding to the persistent closure of the estuary and increase in salinity to 60 ppt [18]. Of the 16 species found over the 4 decadal periods available for this lagoon, *T*. *californianus* was the only one found at every period and the only species present in the most recent time period. *Laevicardium substriatum*, *Leptopecten latiauratus*, *L*. *laciniata*, *Macoma* sp., *T*. *nuttallii*, and *M*. *galloprovincialis* were only found in the late 1960s-mid 1970s; *M*. *nasuta* and *Mactrotoma californica* were found in the late 1980s-mid 1990s; *Laevicardium* sp., *L*. *staminea*, *Tellina* sp., and *M*. *senhousia* were also found from the late 1980s but persisted until the mid 2000s; and *Argopecten ventricosus*, *Donax californicus*, and *Chione* sp. were only found in the late 1990s-mid 2000s.

A total of fourteen native bivalve species and two introduced species (*M*. *senhousia* and *M*. *galloprovincialis*) were found in samples from this lagoon throughout the study period (S1 Table). Of the two introduced species, *M*. *senhousia* was most abundant and persistent. It appeared and peaked in the late 1980s-mid 1990s (up to 24 ind. per 490cm^2^) and remained at lower abundance throughout the late 1990s-mid 2000s (4–5 ind. per 490cm^2^) (S1 Table). However, patches of *M*. *senhousia* were observed fall, 2006, outside of sampling stations in the eastern end of Los Peñasquitos Lagoon. *Mytilus galloprovincialis* was present in the late 1960s, but has since disappeared from surveys.

Mission Bay. Of the 12 species found in Mission Bay throughout this study period, only *M*. *senhousia*, an introduced species, and *T*. *californianus* were present in all four decadal time periods available for this bay (S1 Table). *Musculista senhousia* has generally been the most abundant species, appearing in the 1960s (avg. of 7 individuals per 490 cm^2^), peaking in the late 1980s-mid 1990s (avg. of 2038 ind. per 490 cm^2^ in 1995), and remaining in relatively high abundances through the most recent surveys (avg. of 1 to 57 ind. per 490 cm^2^) (S1 Table). *Leukoma staminea* was only found in the late 1960s-mid 1970s and late 1980s-mid 1990s, (S1 Table). *Chione fluctifraga* and *C*. *californica* were also present in the late 1960s-mid 1970s, but disappeared after that sample period with only *C*. *fluctifraga* reappearing in the late 2000s-present. Both *Chione undatella* and *M*. *nasuta* were present in the late 1980s-mid 1990s, were absent in the late 1990s-mid 2000s surveys, and reappeared in the recent surveys. *Clinocardium nuttallii*, *Lyonsia californica*, *M*. *secta*, and *V*. *philippinarum*, an introduced species, also appeared in the late 2000s-present. The appearance of species in Mission Bay in the most recent time period may, in part, be a factor of a larger sampling effort with 5 sampling events compared with 1–2 in previous time periods. In the 2013 survey, the two introduced species, *M*. *senhousia* and *V*. *philippinarum*, were the two most abundant species in this estuary (57 and 24 individuals per 490 cm^2^, respectively) (S1 Table).

Tijuana River Estuary. Three of the 39 species collected over the 4 decade time periods, *L*. *staminea*, *M*. *nasuta*, and *T*. *californianus*, were present in every period. The natives, *Chione undatella*, *C*. *californica*, *L*. *substriatum*, *N*. *nuttallii*, *S*. *nuttalli*, *Tellina carpenteri*, and *T*. *nuttallii* were found in the late 1960s-mid 1970s up to the late 1990s-mid 2000s, but were absent in the late 2000s-present surveys. Several native and introduced species (*Chione* sp., *Cumingia californica*, *Laevicardium* sp., *Lyonsia californica*, *M*. *senhousia*, *M*. *galloprovincialis*, *Solen rosaceus*) that were found in samples from the late 1980s-mid 1990s were also absent in the late 2000s-present. The lack of detection of these species may in part be due to lower sampling effort in the most recent time period (2 sampling events) than in earlier time periods (9–10 sampling events), however there were also only 2 sampling events during the late 1960s-mid 1970s period when many of the native species were found.

In total, 36 native bivalve species and four introduced species (*Crassotrea gigas*, *M*. *senhousia*, *M*. *galloprovincialis*, *V*. *philippinarum*) were found in Tijuana River Estuary during this study period (S1 Table). *Musculista senhousia* has generally been the most abundant introduced species found in Tijuana River Estuary, appearing and peaking in the late 1980s-mid 1990s (as high as 164 individuals per 490 cm^2^), remaining present but in generally lower abundances in the late 1990s-mid 2000s (14–55 individuals per 490 cm^2^) and afterward disappearing from samples. Although not present in our samples, dense patches of a fourth introduced species, *V*. *philippinarum* were observed in 2013. It was associated with shell hash about 3–5 cm tidal elevation lower than where we sampled for this project.

## Discussion

Examination of historical data spanning thirty to fifty years, in combination with newly-collected data, demonstrated that bivalve communities have undergone dramatic changes at four estuaries located throughout southern California. The only system with no change, Bahía de San Quintín, northern Baja California, was due the continued absence of bivalves in samples. In the late 1960s, only intact shells and no live bivalves were found indicating recent inhabitation and die off [20]. Although we did not quantify abundances of shell material during the 2013 sampling, we noted a striking absence of any sort of intact or nearly intact shells indicating that there has been no apparent recolonization since that mid-century die off.

Throughout southern California over the last 50 years, bivalve diversity fluctuated with booms and busts of introduced species, and ended with similar or lower levels of diversity as compared to the late 1960s-mid 1970s. Community compositions have changed with intertidal losses of several native species and concomitant gains of at least two introduced species. In general, community structure has shifted from the presence of larger, longer-lived bivalve species to a predominance of faster-growing, surface-dwelling smaller species [38]. The appearance and peaks of new species, and the disappearance and, sometimes, reoccurrence of species demonstrated the limitations of using historical data in which data gaps exist, and the importance of long-term and at least semi-regular monitoring of estuarine communities.

### Regional bivalve community changes

#### Declines and losses

Crooks (2001) saw the beginning of declines of native bivalves in Mission Bay, and now we are seeing apparent declines in several systems across the region. Small- to medium-sized native bivalves (e.g., *C*. *californica*, *L*. *substriatum*, *T*. *carpenteri*) that were present in the late 1960s, and at times persisted to the mid 1990s, declined by the late 1990s-mid 2000s and disappeared from the intertidal by the late 2000s. Large natives (e.g., *N*. *nuttallii*, *S*. *nuttalli*, and *T*. *nuttallii)* that were present, and sometimes dominant (e.g., *N*. *nuttallii*), in the late 1960s began to disappear from intertidal samples throughout the 1970s and 1980s. Several of these bivalves (e.g., *C*. *californica*, *L*. *substriatum*, *N*. *nuttallii*, *S*. *nuttalli*, *T*. *nuttallii*) still occur in the subtidal waters of Mission Bay [39,40], and are only reduced or absent from intertidal elevations. The other California estuaries do not have extensive subtidal regions so may not have similar subtidal refuges [41]. Bahía de San Quintín, however, does have extensive subtidal area (ca. 2200 ha, or about 45% of the bay) [31], yet the two study wetlands have remained devoid of bivalves since the 1960s, indicating other factors in play.

While climate change may have contributed to observed trends [15,42], the ranges of most bivalves found throughout this study period extended well beyond the extent of our study locations [38] making it difficult to test for climate related shifts. It is also unlikely that this is the only influential factor [15], as intertidal losses may be the result of ocean inlet restrictions and closings, intertidal sediment deposition, and poor water quality [6,18,21,28,43]. The inlets of most estuaries in southern California are channelized or constrained to allow surrounding urban development including buildings, and both railroad and highway overpasses. These narrow channels commonly fill with sand, reducing tidal exchange with the estuary [44]. The effects of reduced tidal flushing include eutrophication, hypoxia and salinities that vary with time of year [41]. Hypersaline conditions form when closures occur in the warm, dry months, while hyposaline conditions result during cool, rainy months. Closures of the mouth of Los Peñasquitos Lagoon throughout the late 1950s-early 1960s, coupled with discharge of treated sewage effluent, resulted in extremely variable soil and water conditions [45]. Water salinity fluctuated between fresh and hypersaline conditions (60 ppt), water quality declined, and losses of plants (cordgrass; [44]), and bivalves [18] were observed through this period. Nearly annual closures at Los Peñasquitos have continued leaving inhabitants vulnerable to fluctuating, extreme conditions until dredging can occur, and potentially contributing to die-offs and the species poor bivalve community (e.g., [44]). A closure of the mouth of the Tijuana River in 1984 resulted in hypersaline conditions and was associated with reductions or losses of many bivalves, including *S*. *nuttalli* and *N*. *nuttallii*, as well as many other invertebrates [46].

Even without inlet closures, estuaries in this region can experience significant salinity reductions, associated with severe storms. Heavy rains over a 10-day period in 1969 reduced salinities in Mugu Lagoon enough to cause significant mortality of low-salinity intolerant species such as *L*. *substriatum and T*. *californianus* [21]. Losses of bivalves, such as *L*. *laciniata* and *M*. *secta*, occurred in Tijuana River Estuary after catastrophic flooding in 1980 [46]. Such episodic storm events contribute to the temporal and cross-estuary variability of species [21,47,48]. The increased frequency and intensity of storms predicted with climate change [49] may therefore continue to alter the variability and structure of bivalve communities.

Over-harvesting may have also contributed to intertidal bivalve declines especially for large species [43]. Early commercial and sport fish may have reduced populations through direct overharvesting, and through indirect effects of destructive fishing techniques [1,4,5], making populations more vulnerable to disturbance and stress [50]. However, Mugu Lagoon, which has been highly restricted to public access and fishing, has still experienced native bivalve declines.

Habitat loss and associated influences of development may have contributed to intertidal bivalve declines since the 1960s. Well documented is the direct loss of at least 75% of historic estuarine area in this region due to land development [51]. Urban and industrial development increases contaminant levels of coastal waters [52,53,54], clearing of lands surrounding estuaries and adjacent watershed increases sedimentation rates [55], and agricultural and residential development increases nutrient inputs [56], all of which have direct and indirect effects on bivalve communities [57]. The absence of live bivalves, but the presence of many intact shells in the two wetland sites in Bahía de San Quintín in the mid-1960s indicated recent die-offs [21]. Corresponding with the 10–20 years leading up to this finding was expanded land clearing for agriculture, the construction of several motels, and the establishment and operation of a fish cannery on the lowlands surrounding the bay [58]. Although no data exist linking this development to changes in the intertidal, such bursts of development in wetland transition and adjacent upland areas likely have some influence on adjacent estuaries (e.g., [59,60]).

#### Persistence

Several native species were common historically and, despite localized declines and increases, can still generally be found across the region, including *T*. *californianus*, *L*. *staminea*, and *M*. *nasuta*. The regional persistence of these species may be in part attributed to their tolerance to a wide range of sediment textures, with a preference for sandier areas [2,23,39]. Shifts to coarser sediments due to beach nourishment activities have been observed in localized areas throughout the region, which may contribute to the persistence of these species [2,61,62]. The wetland in Mission Bay contained the highest sand content in comparison to the wetlands of Mugu Lagoon, Los Peñasquitos Lagoon, and Tijuana River Estuary [22], which corresponded with the finding of bivalve assemblages characteristic of sandier substrates (*T*. *californianus*, *M*. *nasuta*, *V*. *philippinarum*, *Clinocardium nuttallii*).

Although *L*. *staminea* and *T*. *californianus* were still present in 2013 and suitable sediments existed in all estuaries [22], they were in lower abundances than in past years. Further, average length of these species reduced by half between 1976 and 1986 in the Tijuana River Estuary [23,24,28]. Nearly constant wastewater inflows throughout the 1980s and into the 1990s in the Tijuana River Estuary lowered salinity, and were attributed to declines in size and abundance of *T*. *californianus*, *M*. *nasuta*, and *L*. *staminea* in stations closest to the inflow but not farther away since all stations contained suitable sediments [28]. Similarly, current sediment and water quality conditions in Mission Bay and Tijuana River should have been suitable to support the broadly tolerant, surface dwelling suspension-feeders, *L*. *staminea* and *Chione undatella*, but both were absent from 2013 samples and additional visual surveys of the wetland creeks and tidal flats (T. Talley, personal observation) suggesting other factors at work [2]. One contributing factor may be the increase in both estuaries of *V*. *philippinarum* abundance (this study, [63]), which was associated with declines of *L*. *staminea* in British Columbia [64].

#### Introduced species and bivalve community changes

Introduced species abundances in these estuaries fluctuated through time. *Musculista senhousia*, a short-lived and fast-growing invader, had a “boom and bust” population cycle in Mission Bay and, along with *M*. *galloprovincialis*, in Tijuana River Estuary (S1 Table) [2]. Both species were common in the early 1990s, peaked in abundance in the mid 1990s and declined again by the early 2000s (S1 Table) [22]. During the peak of *M*. *senhousia* in Mission Bay in the 1990s there was a decline in surface dwelling species (e.g., *Chione fluctifraga*, *L*. *staminea*) but not deeper dwelling species (*M*. *nasuta*, *T*. *californianus*) [2]. However, despite the post-boom declines of these introduced species, abundances of the surface dwellers have not returned to their previous levels and there remains a greater number of introduced species, such as *M*. *senhousia* and *V*. *philippinarum*, in these estuaries than in the mid-late 1960s. Indeed, *V*. *philippinarum* has increased in abundance during the last five years in Mission Bay [22, 63]. This species was also present in 2013 in Mugu Lagoon and was observed in dense patches in Tijuana River Estuary outside of sampling stations. This moderately fast growing *V*. *philippinarum* (mature at 1–2 cm, 1.5–2 years) [65] has been reported to dominate some intertidal regions of San Francisco Bay and Tomales Bay in northern California, and coastal British Columbia, Canada [64]. Declines of *L*. *staminea* in the Pacific Northwest of North America have been linked with the expansion of *V*. *philippinarum*, which has a similar filter feeding lifestyle and sediment preference to the native [64,66]. Faster growth rates allow the invader to outcompete the native for food [64,67]. Further, introduced species, like *V*. *philippinarum* found in this study and *Nuttallia obscurata* found in British Columbia, may alter intertidal communities by serving as an accessible food source for predators [66,68] thereby increasing the predator’s population and potential predation pressure on all prey species. While additional work is needed to verify interactions from this study, increased occurrence of *V*. *philippinarum* was observed in estuaries exhibiting declines in *L*. *staminea* (Tijuana River Estuary, Mission Bay, Mugu Lagoon).

## Conclusion

Intertidal bivalve communities throughout the southern California and northern Baja California region displayed trends of native species declines, and/or gains of introduced species over the last 30–50 years. Regional climate change effects may have contributed to general, regional trend [15, 42], but the extent of these influences are uncertain due to data gaps and the majority of species being fairly ubiquitous throughout the study region. The ecological history, structure (e.g., depth and size), and location (e.g., local climate, watershed development effects) of each estuary likely contribute to the maintenance of relatively distinct communities despite shifts within estuaries with time [21,69,70]. Both episodic events, such as El Niño events, storms and inlet closures, and gradual environmental changes, such as sand intrusion and species introductions, likely influence communities and contribute to spatial and temporal variability in bivalve communities within and between estuaries [2,6,21]. The ecosystem functioning and services of these systems also likely change as bivalve communities shift from deeper-dwelling, larger taxa towards a predominance of surface-dwelling, smaller taxa [8,11,12,71]. This has implications for how future changes associated with climate change, land use, and water use will influence the structure and services provided by estuaries, and is an area in need of investigation.

Predicting change is the challenge. Historical data are valuable for understanding patterns and offering insights into drivers of change and subsequent research priorities, but there are limitations to this approach such as the gaps in data through time and the availability of explanatory environmental data. One clear recommendation from our work is the need for consistent long-term monitoring programs, which will allow for more robust assessment of changes as well as management actions such as rapid response to potentially problematic invaders. Also, based on our findings, we recommend that future research include the quantitative testing of the environmental and demographic factors potentially driving declines and/or limiting recovery of native bivalve communities. In particular, testing the relative effects of environmental conditions, such as water quality and contaminants, sedimentation and freshwater flows, and interactions with introduced species, on bivalve communities would help to determine whether current day estuaries are even suitable anymore for supporting lost and declining natives. Studies of intrinsic influences on bivalve recovery may include a focus on reproductive, dispersal or recruitment limitation (e.g., Allee effects), and could be explored in estuaries with refuges, such as extensive subtidal areas. It is this understanding of the mechanisms that underlying change that is crucial if we are to predict the outcomes of ecosystem and bivalve restoration, the success of aquaculture, and in general how ecosystems will change into the future.

## Supporting Information

S1 Table Average bivalve abundances (S.E.) at each estuary for the time periods used in this study. (XLSX)
