# Correction: Spatial and Temporal Examination of Bivalve Communities in Several Estuaries of Southern California and Northern Baja California, MX

**DOI:** 10.1371/journal.pone.0151727

**Published:** 2016-03-11

**Authors:** Anai Novoa, Theresa S. Talley, Drew M. Talley, Jeffrey A. Crooks, Nathalie B. Reyns

There are errors in the Funding section and Acknowledgements section. The complete, correct Funding statement is as follows:

No external funding was received for this study. Some resources (supplies and research assistance) were donated by T.S. Talley through California Sea Grant Project A/EA-AR-37, Ocean Discovery Institute, and D.M. Talley and N. Reyns through Environmental and Ocean Sciences at University of San Diego. The statements, findings, conclusions and recommendations are those of the researchers and do not necessarily reflect the views of the aforementioned organizations. The funders had no role in study design, data collection and analysis, decision to publish, or preparation of the manuscript.

The complete, correct Acknowledgements section is as follows:

We are grateful to the University of California Natural Reserve System, Tijuana River National Estuarine Research Reserve, U.S. Fish and Wildlife Service, Torrey Pines State Park, Ocean Discovery Institute, as well as Mark Page, Steve Schroeter, Andres Deza, and Justin Hoestery for site access and/or use of data. Thanks to the field sampling assistance of Rosa Calvario, Marlem Rivera, Carla Pisbe, Christine Whitcraft, Larisa Chavez, Liz Lopez, Nathalie Reyns’ 2009–2011 Marine Community Ecology classes at University of San Diego, and Paul Detwiler’s 2013 Marine Science class at San Diego Mesa College. We also thank Erik Thuesen and two anonymous reviewers for helpful comments that improved this manuscript.
